# Genetic diversity and dynamics of plum pox virus populations in the alternative host American plum

**DOI:** 10.1007/s00705-025-06502-3

**Published:** 2026-01-26

**Authors:** Tamara D. Collum, Andrew L. Stone, Elizabeth E. Rogers

**Affiliations:** 1 Agricultural Research Service, USDA, Appalachian Fruit Research Station, 2217 Wiltshire Rd, Kearneysville, WV 25430 USA; 2 Agricultural Research Service, USDA, Foreign Disease-Weed Science Research Unit, Frederick, MD USA

## Abstract

Plum pox virus (PPV) is a serious viral threat to stone fruit trees worldwide. Wild *Prunus* species including American plum (*Prunus americana*) can serve as sources of inoculum. High-throughput sequencing was used to characterize PPV populations in American plum and peach after aphid inoculation and after two cycles of cold-induced dormancy (CID). A significant decrease in the number of sequence variations in the PPV genome was observed after CID in American plum, but not in peach. Seventeen were identified as unique to American plum, while eight were unique to peach. These findings provide insight into the genetic diversity of PPV in a potential reservoir host.

**Supplementary Information:**

The online version contains supplementary material available at 10.1007/s00705-025-06502-3.

Plum pox virus (PPV), the causative agent of the disease sharka, is the most serious viral threat to stone fruit trees worldwide. Since its first detection on plum in Bulgaria around 1917, the global cost of PPV has been estimated to exceed $15 billion [1]. The name sharka is the Bulgarian word for pox. In addition to the chlorotic rings and spots that gave plum pox its name, symptoms can include vein clearing, leaf distortion, fruit deformation and discoloration, reduced fruit quality, and premature fruit drop. Ten PPV strains are currently recognized based on sequencing and phylogenetic analysis. The host range of PPV is overlapping and only partially strain-specific. PPV-D is the prevalent strain of PPV and is globally widespread [1]. Historically, PPV-D has been associated with plum infections, but it can also infect peach, nectarine, apricot, and almond [1–4].

In the United States, PPV-D was first found in 1999 in Pennsylvania. Although the Pennsylvania isolates of PPV-D have a large host range under experimental conditions, most PPV infections in the United States have been found in cultivated peach (*Prunus persica*) and plum (*P. domestica*) [5, 6]. However, the native North American *P. americana,* commonly called American plum, has the potential to act as a reservoir host for Pennsylvania isolates of PPV-D and may potentially serve as a source of infection for important commercial crops such as peach, but little is known about PPV population dynamics in this alternative host [7]. In 2019, the U.S. Department of Agriculture declared that PPV had been eradicated in the United States [8]. PPV is also considered eradicated in Nova Scotia, Canada, but it is still present in the Niagara Region in Ontario, Canada [9].

PPV is a member of the genus *Potyvirus* in the family *Potyviridae*. The PPV genome is typical for a potyvirus and is comprised of a positive-sense single-stranded RNA containing a large open reading frame (ORF) that encodes 10 proteins (P1, HC-Pro, P3, 6K1, CI, 6K2, VPg, NIa-Pro, NIb, and CP) and an additional small overlapping ORF called PIPO that is translated as fusion product, P3N-PIPO, by slippage of the viral RNA-dependent RNA polymerase [2, 10].

Viral genetic diversity plays a significant role in virus-host interactions and adaptation to new environments [11–13]. RNA virus populations are considered to exist as ‘quasi-species’ or groups of closely related genomic sequences [14]. Sequence diversity is increased by the error-prone nature of viral RNA polymerases, which lack proofreading activity, and the large population sizes in infected hosts. The long-lived nature of perennial fruit trees can also lead to the evolution of complex viral populations [15]. Diversity can be reduced through selection and bottlenecks, which can occur during transmission by aphid vectors or movement within a host [16, 17]. High-throughput sequencing technology has improved our ability to characterize genetic variation within virus populations and detect viral variants that occur at low frequencies [18–20]. To date, research examining the genetic diversity of PPV-D has primarily relied on analysis of consensus sequences and has not been performed in the reservoir host American plum.

In this study, we used high-throughput sequencing to identify variations in the PPV-D genome in American plum and peach (*P. persica* cv. GF305). PPV-D Penn39 was maintained both by grafting and aphid transmission to healthy *Prunus persica* cv. GF305 peach seedlings for approximately 20 years in the U.S. Department of Agriculture (USDA)-Agricultural Research Service plant BSL3 containment facility at Ft. Detrick, Maryland. All trees were grown under standard greenhouse conditions (14 h daylight, temperatures 24 to 26 °C daytime, 20 °C nighttime) and vernalized every 3 to 6 months for a 60- to 90-day cold-induced dormancy (CID) period in a 4 °C dark cold box. Healthy GF305 peach and American plum trees, 1 to 2 years old, were inoculated using green peach aphids (*Myzus persicae)* placed on detached symptomatic leaves from a Penn39-positive GF305 peach tree as described previously [7]. Leaf punches were taken from systemic leaves from four individual trees of each species at 60 days after initial aphid infection and at 60 days post CID2. Each tree served as a biological replicate with 10 systemic leaf punches pooled per tree. Total RNA was isolated using a Plant RNeasy Kit according to the manufacturer’s specifications (QIAGEN, Germantown, MD) and sent to Azenta Life Sciences (South Plainfield, NJ) for poly-A selection and cDNA library preparation. Libraries were barcoded and sequenced on an Illumina HiSeq platform, yielding between 12 and 129 million 150-bp paired-end reads per sample (Supplementary Table S1). Sequencing data are available under NCBI bioproject accession no. PRJNA881753 (https://www.ncbi.nlm.nih.gov/bioproject/881753).

The total reads obtained from each sample were trimmed and filtered based on their quality score and mapped to the sequence of the inoculated strain, PPV-D Penn39 (PX208212) using CLC Genomics Workbench. Analysis of variants was carried out using the CLC Genomics Workbench low-frequency variant detection tool with the following parameters: Required significance = 1.0%, Min coverage = 10, Min count = 2, Min frequency = 1.0, Ignore non-specific matches = Reads. The Illumina sequencing error rate has been estimated to be approximately 0.1% [21], so a cutoff of 1.0% was applied for identifying PPV variants, which is tenfold higher than what would be expected from sequencing errors. After filtering, a total of 813 unique variations were identified from all samples and time points, 74% of which were nonsynonymous. Read counts and mapping statistics for all samples are available in Supplementary Table S1, and all of the variations identified in this study can be found in Supplementary Table S2.

Nearly complete genome sequences could be assembled from each sample, and these were 99.69–99.83.69.83% identical to the Penn39 source genome. A maximum-likelihood phylogenetic tree (Fig. 1) was constructed from these consensus sequences and other complete PPV-D genome sequences using Geneious Prime v. 2025.2.1 and IQTree v. 2.4.0 as described previously [5]. All 16 consensus sequences formed a well-supported clade that was sister to the Penn39 source and within a larger clade of sequences from Pennsylvania. It is notable that the four American plum post-CID2 samples grouped together, which reflects the genome changes that occurred over two growth and dormancy cycles in the novel American plum host.

**Fig. 1:**
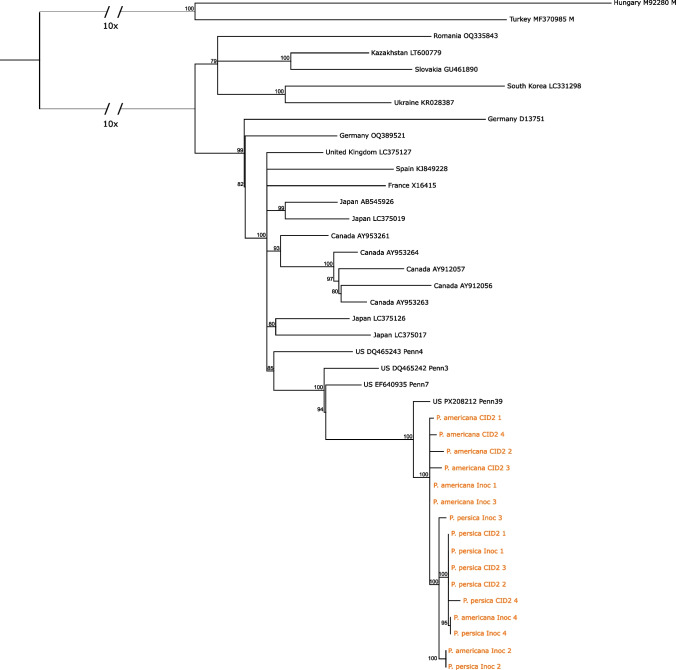
Maximum-likelihood phylogenetic tree of selected PPV-D isolates. The tree is rooted on two M isolates (at the top of the tree). Lines connecting the D and M clades have been shortened to 10% of their original length to facilitate display on a single page. Each isolate is labeled with its country of origin and GenBank accession number. Consensus sequences from this study are labeled in orange text

The number of variations observed per sample ranged from 36 to 185 for peach and 37 to 183 for American plum (Supplementary Table S1). In peach, there was no significant difference between the total number of variations observed in post-inoculation samples and samples collected after CID2. However, in American plum, the total number of variations observed decreased after CID2 (Fig. 2). We did not observe a relationship between the total number of PPV reads and the number of variations observed.

**Fig. 2:**
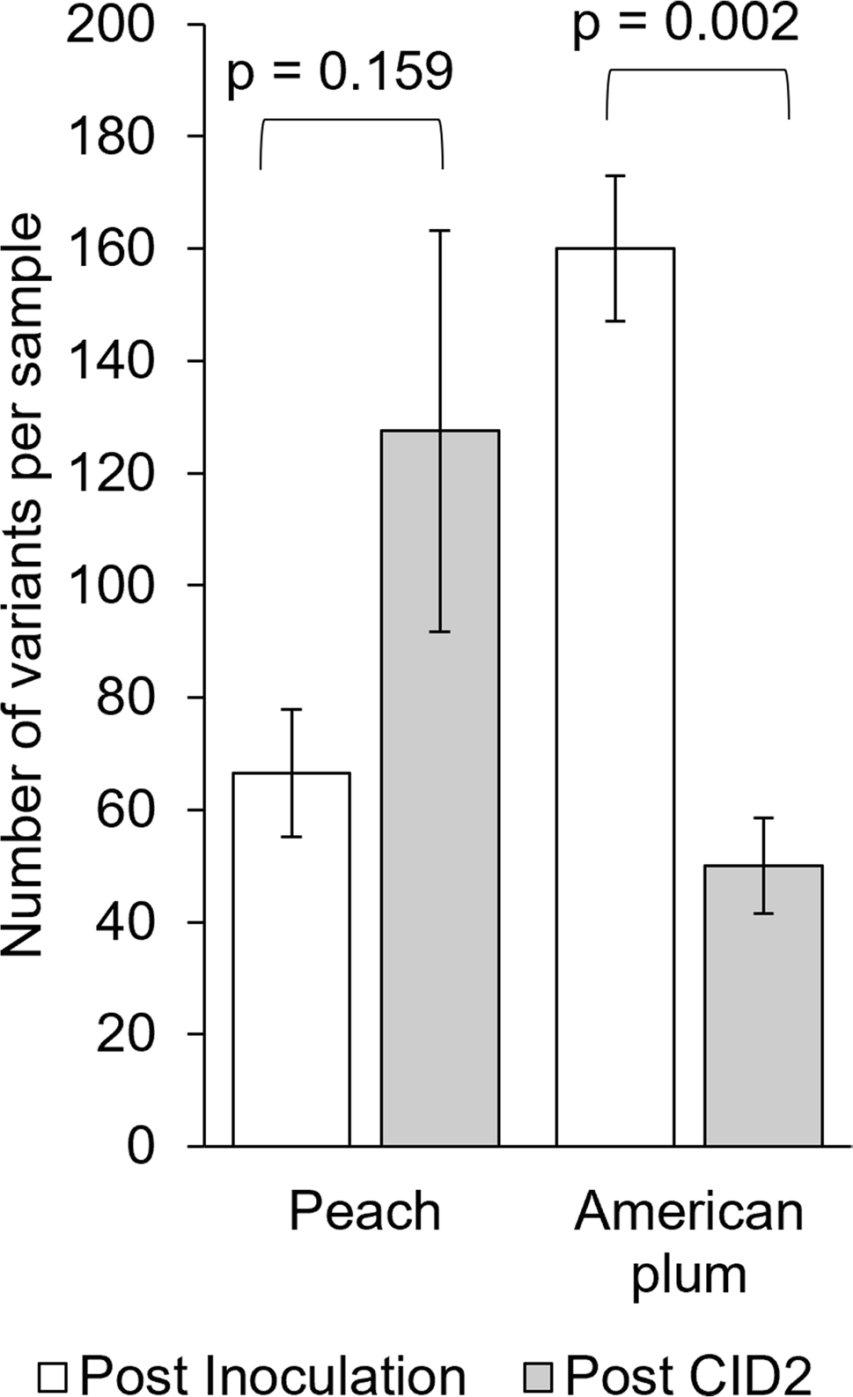
Comparison of the number of nucleotide variations in the PPV genome in infected peach and American plum leaves. Samples were collected after inoculation and after the second round of cold-induced dormancy (CID2). The observed variations include nucleotide polymorphisms, indels, and SNPs. Bars represent the mean ± SE of four biological replicates. Statistical analysis was performed using a two-tailed Student’s *t*-test

Of the 813 total variations observed, 267 (33%) were unique to peach samples and 437 (54%) were unique to American plum samples. Ninety and 93% of the host-specific variations (239/267 for peach and 406/437 for American plum) were observed only in one sample (Supplementary Table S2). To further analyze differences in PPV populations between hosts, we only considered variations that were observed in at least four of the 16 samples. This included 103 variations, 43 of which (42%) were nonsynonymous and 101 of which were single-nucleotide variations (SNVs). An insertion at position 2907 was detected in 15 of the 16 samples. This insertion, an ‘A’ at position 2907, is part of the slippage site preceding the PIPO ORF and was observed at a frequency of 4.9% in peach and 5.6% in American plum (Supplementary Table S3). This is higher than the 1.6% slippage rate reported previously for *P. domestica* ‘Jojo’ infected with PPV-D and PPV-Rec [22] but similar to the 4.0% rate observed in *P. domestica* ‘Stanley’ and ‘President’ infected with PPV-D [19]. A deletion at position 7786 was observed in two American plum samples and three peach samples at a frequency of 1.01–5.31.01.31%, and this deletion results in a premature stop codon in the NIb coding region.

Of the 101 SNVs, 14 occurred in all 16 trees at a frequency of >67%. These mapped to the P1, HC-Pro, P3, CI, 6K2, VPg, NIb, and CP coding regions (Fig. 3A). A3296T in P3 and A7400G in NIb were the only two SNVs not observed in other PPV-D isolates based on the analysis of consensus sequences of complete PPV genomes retrieved from GenBank (Supplementary Table S4). Only three SNVs (A3296T in P3, A6279G in VPg, and A8892G in CP) were nonsynonymous; these three, along with most other SNVs, were observed in other PPV-D isolates.

**Fig. 3:**
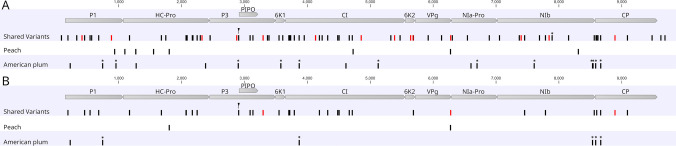
(**A**) Genome positions of shared variations and variations unique to peach and American plum observed in at least four of the 16 samples. (**B**) Genome positions of shared nonsynonymous variations and nonsynonymous variations unique to peach and American plum observed in at least four of the 16 samples. Variations observed in all 16 samples are colored red. * indicates that the variation was significantly more common in American plum than in peach when using Fisher's exact test (FDR *p*-value < 0.05)

Eight of the 101 SNVs were observed only in peach and 17/101 SNVs were observed only in American plum (Fig. 3A). Fisher's exact test was used to identify variations that were significantly more common in American plum than in peach samples (FDR *p*-value < 0.05). These included 12 of the 17 SNVs identified as unique to American plum and one shared SNV at position 7892 that was observed in all eight American plum samples and in only one peach sample (Fig. 3). No variations were significantly more common in peach than in American plum, as all of the variations that were unique to peach occurred only in four or five of the eight peach samples.

SNVs observed only in peach mapped to the P1, HC-Pro, CI, VPg, and NIb coding regions. Two SNVs that were unique to peach were nonsynonymous: I1803F in HC-Pro and E6276L in VPg (Fig. 3B). SNVs that were only observed in American plum mapped to the P1, HC-Pro, P3, 6K1, CI, NIa-Pro, NIb, and CP coding regions (Fig. 3A). Only six of the SNVs unique to American plum were nonsynonymous. These include A228T and N744D in P1, M3870V in CI, D8535N in NIb, and E8585D and P8665L in CP (Fig. 3B). With the exception of P8665L, these SNVs have been observed in other PPV-D isolates.

In conclusion, the total number of PPV variants observed in American plum decreased significantly post-CID2, but the total number of PPV variants observed in peach did not change. In contrast, there were more host-specific PPV variations in American plum than in peach (17 vs. eight). While further experiments will be needed to determine the role the identified variations may play in viral biology, this study provides new information on the genetic variation of PPV and its population dynamics in the alternative host American plum.

## Supplementary Information

Below is the link to the electronic supplementary material.

Supplementary file1 (XLSX 11 KB)

Supplementary file2 (XLSX 152 KB)

Supplementary file3 (XLSX 10 KB)

Supplementary file4 (XLSX 16 KB)

## Data Availability

The dataset analyzed in the current study is available under NCBI Bioproject accession no. PRJNA881753, https://www.ncbi.nlm.nih.gov/bioproject/881753.
